# Distinct mechanisms by which two forms of miR-140 suppress the malignant properties of lung cancer cells

**DOI:** 10.18632/oncotarget.26356

**Published:** 2018-11-23

**Authors:** Valentina Flamini, Ed Dudley, Wen G. Jiang, Yuxin Cui

**Affiliations:** ^1^ Cardiff China Medical Research Collaborative, School of Medicine, Cardiff University, Heath Park, Cardiff, CF14 4XN, UK; ^2^ Swansea University Medical School, Swansea University, Singleton Park, Swansea, SA2 8PP, UK

**Keywords:** microRNA, lung cancer, metabolism, RNA-Seq, signalling pathway

## Abstract

In this study we attempted to determine the molecular mechanisms underlying the two mature products of pre-miR-140 (3p and 5p) in malignant properties of lung cancer cells. The differential expression of the two forms of miR-140 in both NSCLC tissues and cell lines was determined by quantitative real-time PCR (qRT-PCR). The effects of the miR-140 mimics on the malignant properties of lung cancer cells were evaluated using invasion assay, adhesion assay, tubule formation assay and metabolite profiling. Biotin-miRNA pulldown and transcriptome profiling by RNA-seq were utilized to distinguish their mRNA targets of the miR-140 strands. Their downstream signalling pathways were unveiled using a high-throughput antibody array. Although both strands of the miR-140 are downregulated in the NSCLC, miR-140-3p is more predominant compared to miR-140-5p in lung cancer cell lines. Both miR-140 mimics suppress the invasion of lung cancer cells and the inhibitory effect of the miR-140 on adhesion is cell-dependent. Tumor conditioned media from A549 cells after treatment with miR-140-3p mimic reduce the tubule formation ability of the endothelial cells. Metabolite profiling indicates the alteration of glycine in both lung cancer cells following treatment with miR-140 mimics. The data from the RNA-sequencing and antibody array indicate that two miR-140 strands present different targeting and signalling profiles despite the existence of mutual targets such as IGF1R and FOS. In conclusion, two forms of miR-140 both suppress the malignant properties of lung cancer cells but through distinct and multiple mechanisms.

## INTRODUCTION

MicroRNAs (miRNAs or miRs) are small non-coding RNAs that prevent the translation of their mRNA targets into proteins. Aberrant expression levels of miRNAs are associated with many malignancies, including lung cancer, suggesting their roles as either oncogenes or tumour suppressors [1]. MiR-140 precursor is located on chromosome 16q22.1 and produces two mature single strands named miR-140-3p and miR-140-5p. They were fist studied in congenital malformations, as changes in their expression levels during the embryogenesis leads to some serious developmental defects [2]. During miRNA biogenesis, the duplex of the two mature miRNA strands is produced from the hairpin miRNA precursor by Dicer cleavage. Then, a following strand selection step determines which mature miRNA acts as guide in the mature miRNA-mediated silencing complex (miRISC). However, previous studies show that miR-140-3p and 140-5p are both downregulated in different cancer cells and tissues compared to their normal counterparts, including lung, thus suggesting their roles as tumour suppressors [3–6].

Lung cancer is the most frequent cancer worldwide, with approximately 1.8 million new cases in 2012 (12.9% of the total tumour cases) [7]. Within the two types according to the morphological differences of the lung cancer cells, Non-Small Cell Lung Cancer (NSCLC) accounts about 85-90% of all cases and has a high tendency for developing distant metastases, while Small Lung Cancers (SCLC) comprises about 10-15% of the lung cancer cases [8].

We previously demonstrated the anti-tumour effect of miR-140-5p on NSCLC lung cancer cells [1]. In this study, the role of its sister strand miR-140-3p was further investigated in NSCLC in comparison with miR-140-5p. Following the evaluation of its expression in a NSCLC patient cohort, miR-140-3p was investigated for its effect on the malignant properties of A549 and SK-MES1 cell lines. The effect of tumour conditioned media (TCM) from lung cancer cells treated with miR-140 mimics on the ability of *in-vitro* angiogenic capacity of the primary endothelial cells (i.e. HUVECs) was also investigated. To explore the targeting and mechanisms of the miR-140 strands in a global manner, the pulldown gene targets by biotin-miRNA mimics were analysed by Ion Proton RNA sequencing, which were integrated with the proteomic profile from Kinex™ Antibody Microarray with 878 antibodies embedded.

## RESULTS

### MiR-140-3p is downregulated in NSCLC tissues and lung cancer cell lines

**Figure 1:**
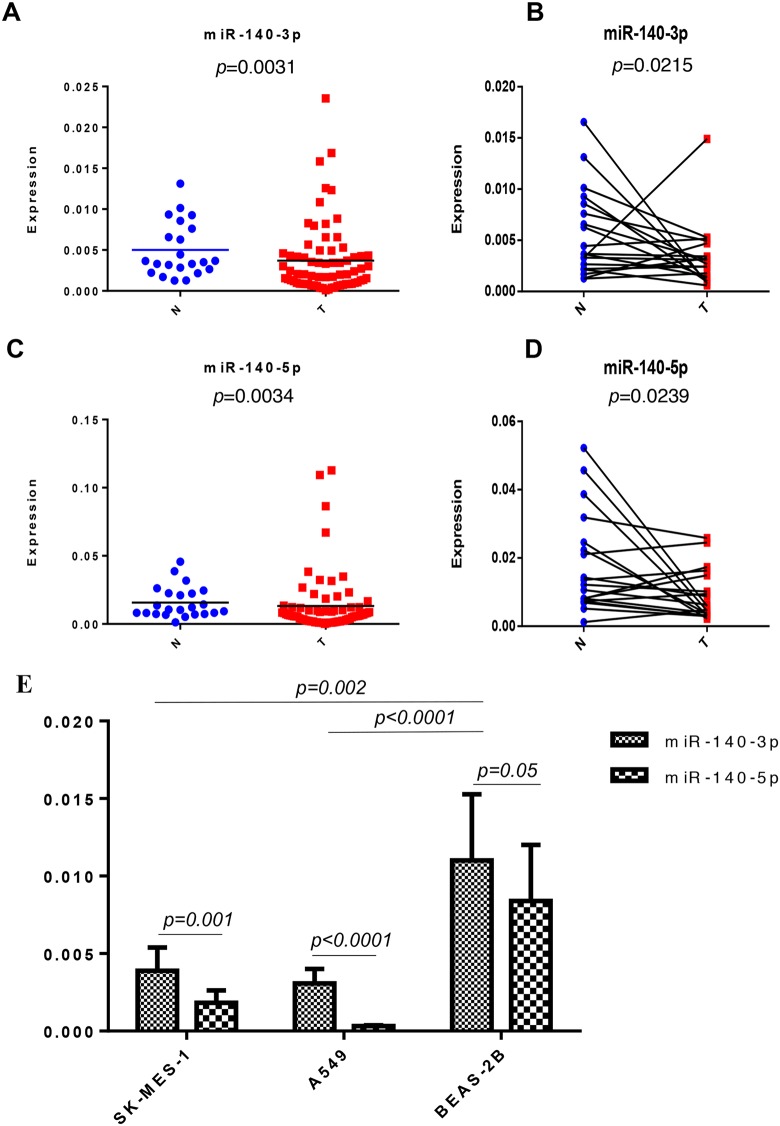
Expression of miR-140-3p in the NSCLC cohort and lung cell lines **(A)** Expression of miR-140-3p in non-paired adjacent-normal (N) and tumour (T) tissues. **(B)** Expression of miR-140-3p in paired adjacent-normal (N) and tumour (T) tissues. **(C)** Expression of miR-140-5p in non-paired adjacent-normal (N) and tumour (T) tissues. **(D)** Expression of miR-140-5p in paired adjacent-normal (N) and tumour (T) tissues. **(E)** Expression of miR-140-3p in two lung cancer cells (SK-MES-1 and A549) and BEAS-2B lung epithelial cells.

In the lung cancer cohort we have obtained, there was lower level of miR-140-3p expression in both unpaired (p=0.0031, Figure 1A) and paired tissues (p=0.0215, Figure 1B) compared to the adjacent tissues. Similarly, we also observed lower level of miR-140-5p expression in both unpaired (p=0.0034, Figure 1C) and paired tissues (p=0.0239, Figure 1D) compared to the adjacent tissues. We further investigated the expression of the both mature miR-140 strands in lung cancer cell lines. In accordance with the finding in lung cancer tissue, we found that the expression levels of both miR-140-3p and miR-140-5p were significantly downregulated in the both SK-MES-1 (p=0.002) and A549 (p<0.0001) lung cancer cells compared to the normal lung epithelial cells (BEAS-2B). Also, there were higher levels of miR-140-3p than miR-140-5p produced in SK-MES-1 (p=0.001) and A549 (p<0.0001), whereas a differential expression of these two strands of miR-140 was not observed in BEAS-2B (Figure 1C).

### MiR-140-3p reduces the invasion ability of NSCLC *in vitro*

**Figure 2:**
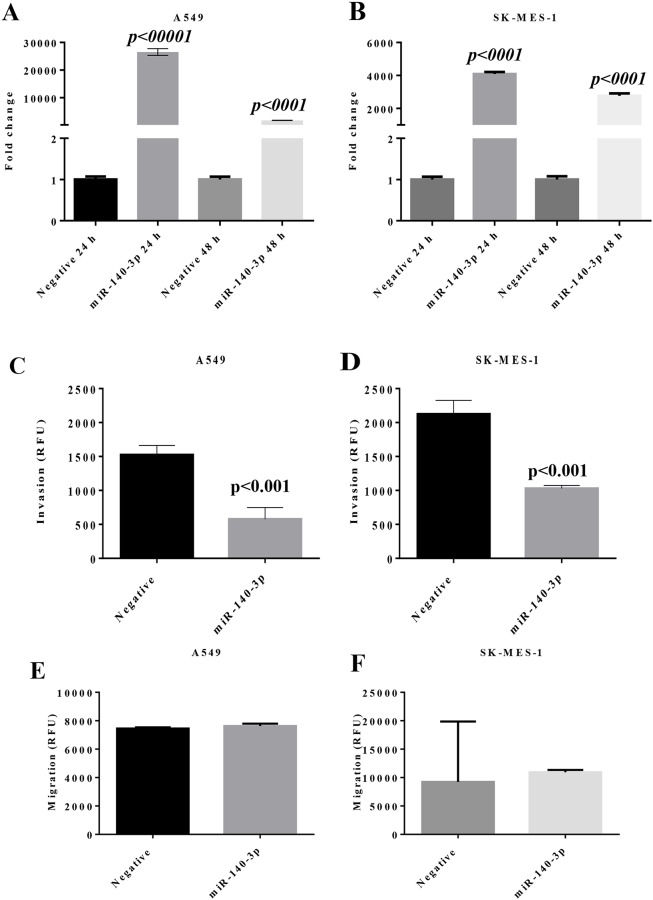
Change of miR expression and invasion properties in lung cancer cells after transfection with miR-140-3p mimic **(A)** Expression of miR-140-3p in A549 cells. **(B)** Expression of miR-140-3p in SK-MES-1 cells. **(C)** Transwell invasion property of A549 cells with response to miR-130-3p mimic. **(D)** Transwell invasion property of SK-MES-1 cells with response to miR-130-3p mimic. **(E)** Transwell migration property of A549 cells with response to miR-130-3p mimic. **(F)** Transwell migration property of SK-MES-1 cells with response to miR-130-3p mimic. Data are presented as mean + SEM, three individual experiments were undertaken in triplicate, Student's *t*-test was used to assess significance.

To investigate the function of miR-140-3p with comparison with miR-140-5p in lung cancer cells, miRNA-140 mimics and a scramble negative control were transfected into lung cancer cells. As shown in Figure 2A, after transfection, the fold change of miR-140-3p levels in A549 cells was 26,508 (p<0.0001 vs Negative mimic) after 24 h and 1737 (p<0.0001) after 48 h. Similarly, the fold change of miR-140-3p levels in SK-MES-1 cells was 4,084 (p<0.0001) after 24 h and 2,757 (p<0.0001) after 48h. In both lung cancer cell lines, following transfection, the levels of miR-140-3p were significantly higher at 24 h than 48h, indicating the dynamic change of the miRNA mimics.

A transwell invasion assay was performed to evaluate whether miR-140-3p exerted any effect on the invasion property of lung cancer cells. As shown in Figure 2C and 2D, transfection with miR-140-3p mimics led to the reduced invasion properties in A549 and SK-MES-1 cells, with p<0.001 in both cell lines compared to the negative mimic controls. In contrast, there was no effect of miR-140-3p on migration of the lung cancer cell lines (Figure 2C). Also we did not observe any change in cell cycle and proliferation after the treatment with miR-140-3p (Supplementary Figure 1).

After the lung cancer cells were treated with the miR-140-3p and miR-140-5p mimics, respectively, we also evaluated the cellular apoptosis level by Annexin V-FITC and propidium iodide (PI) staining. As shown in Supplementary Figure 2, there was no induction of apoptosis following the treatments compared to the negative mimic control.

### The two strands of miR-140 alter the metabolite profile of lung cancer cells in a similar way

**Figure 3:**
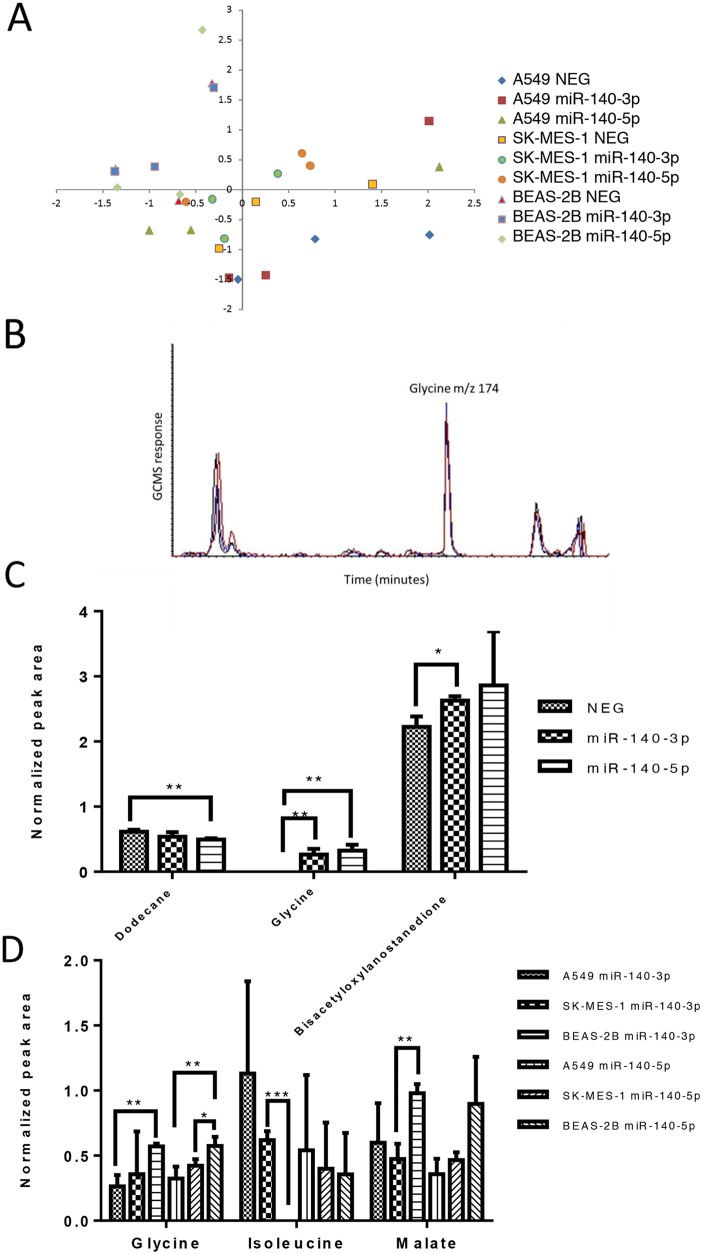
The two strands of miR-140 have minor impact upon the metabolite profile of lung cancer cells Lung cancer cells (A549 and SK-MES-1) and BEAS-2B lung epithelial cells were treated with miRNA mimics for 24 hours before cell samples were collected for metabolite analysis using Mass spectrometry (GC-MS). **(A)** A principle component analysis of the metabolite data set for the three cell lines without treatment (NEG) and with either miRNA mimic. **(B)** Example GCMS chromatogram of m/z 174 representing glycine levels in the control (NEG, black line) and after treatment with miR-140-3P (red line) and miR-140-5P (blue line). **(C)** Normalized peak area of metabolites which differed in level in A549 cells dependent on treatment. **(D)** Normalized peak area of metabolites which differed in level dependent on the cell line studied. Data are presented as mean + SEM, experiments were undertaken in triplicate, *T*-test and Mann Whitney tests were used to assess significance among multiple groups with ^*^p<0.05, and ^**^p<0.01.

To understand whether the treatment with the miR-140 mimics have an effect on the metabolism of lung cancer cells, the cells, following treatment, were subjected to GC-MS metabolite analysis. Within the analysis, 131 metabolite signals were detected and compared following normalisation to the internal standard. As shown in Figure 3A, the principle component analysis using the full metabolite profile does not discriminate either of the cancer cell lines used or the normal lung cell line from each other or allow for separation of cell lines based upon treatment with a miRNA mimic. This suggests that at the global level there is little impact upon the major metabolites identified in this analysis. Upon performing statistical analysis upon each individual metabolite level (rather than studying the total profile via principle component analysis) a few metabolites were identified as generating P values less than 0.05, inferring a statistical difference in levels during the analysis. However, when studying large metabolomics datasets with a limited number of replicates, these differences (although interesting and providing significant initial P values) should be treated as preliminary as multiple hypothesis testing corrections on this scale often reduce their significance. For the A549 cell line only, there was a difference detected in three metabolites based upon treatment with an miRNA mimic, Figure 3B shows an extracted ion chromatogram for m/z 174 (representing the amino acid glycine), which is at very low levels in the A549 cell line when untreated, but elevated following treatment with either miRNA mimic. Only the A549 cells exhibited a difference in metabolite levels based upon treatment (compared to the untreated control cells (NEG)) and these metabolites and their levels are represented in Figure 3C. The increase in glycine levels occurs regardless of which mimic is used (P< 0.01) whilst the change in levels of dodecane and the lanostanedione derivative differ dependent upon treatment with miR-140-5p or miR-140-3p. The other cell lines did not demonstrate a statistically significant difference in metabolite profile dependent upon treatment alone. Further analysis sought to study whether the different cell lines used responded differently to either of the miRNA mimics (Figure 3D) and this analysis demonstrated that the vast majority of metabolites did not vary between cell lines with or without treatment, however glycine was shown to differ in level between A549 cells and BEAS-2B cells following treatment with either mimic (P<0.01) and also between SK-MES-1 and BEAS-2B cells, but only following treatment with miR-140-5p (P<0.05). Malate levels were only identified as differing between SK-MES-1 and BEAS-2B following treatment with miR-140-3p (P<0.01).

### The TCM from cancer cells transfected with miR-140 mimics inhibit the tubule formation in HUVECs

**Figure 4:**
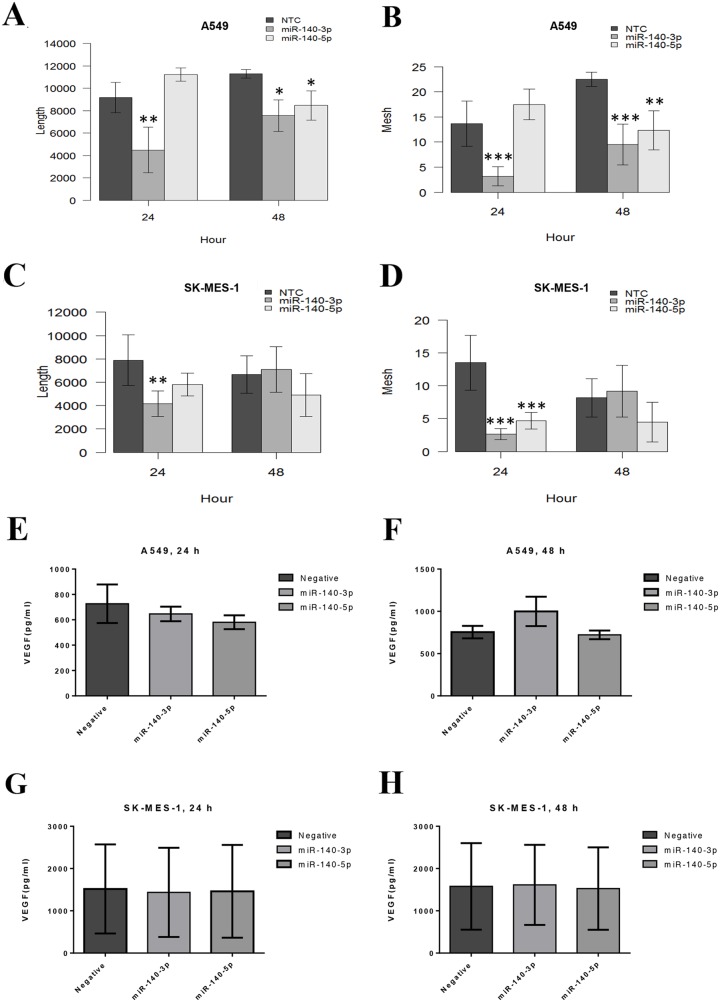
Tubule formation ability of the primary endothelial cells HUVECs co-cultured with TCM of lung cancer cells treated with miR-140-3p and 140-5p mimics HUVECs were seeded onto a layer of Matrigel with completed growth medium supplemented with 40% TCM from miR-treated lung cancer cells for 24 and 48 hours, respectively. The tubule formation was monitored in real-time by using the EVOS Cell Imaging System. Images were taken every 30 minutes for 6 hours. **(A)** Total length of the tubule structure form by HUVECs in the presence of TCM from A549. **(B)** Number of closed mesh structure formed by HUVECs in the presence of TCM from A549. **(C)** Total length of the tubule structure form by HUVECs in the presence of TCM from SK-MES-1. **(D)** Number of closed mesh structure formed by HUVECs in the presence of TCM from SK-MES-1. Data are presented as mean + SEM, three individual experiments were undertaken in triplicate, Kruskal-Wallis-test was used to assess significance with ^*^p<0.05, ^**^p<0.01 and ^***^p<0.001. **(E-H)** Secreted protein level of VEGF in TCM from the two lung cancer cells after treatment with miRNA mimics for 24 and 48 hours, respectively.

As shown in Figure 4, the TCM from A549 cells treated with miR-140-3p for 24 hours reduced the ability of HUVECs to form vessels including length of tubule branch (p<0.01, Figure 4A) and number of vessel-like meshes (p<0.001, Figure 4B). Likewise, the TCM from SK-MES-1 cells treated with miR-140-3p for 24 hours also reduced the angiogenic ability of HUVECs including tubule length (p<0.001) and vessel-like mesh (p<0.0001) (Figure 4C and 4D). The TCM from A549 cells treated with miR-140-3p for 48 hours also reduced the ability of HUVECs to form vessels including length of tubule branch (p<0.05, Figure 4A) and number of vessel-like mesh (p<0.001, Figure 4B). However, the 48-h CM from SK-MES-1 treated with miR-140-3p did not show any effect on tubule formation of SK-MES-1. The 24-h TCM from A549 after miR-140-5p treatment did not show any effect on tubule formation, however 48-h TCM from A549 after miR-140-5p treatment inhibited the tubule formation of HUVECS including the tubule length (p<0.05) and the meshes number (p<0.01). The 24-h TCM from SK-MES-1 after miR-140-5p treatment reduced the tubule meshes number (p<0.001). However, the 48-h TCM from SK-MES-1 after miR-140-5p did not show any effect on tubule formation. The representative images of the tubule formation of the HUVECs co-cultured with the TCM of lung cancer cells treated with miR-140-3p and 140-5p mimics were shown in Supplementary Figure 3.

We further investigated whether the inhibitory effect to miR-140 mimics was due to any change in VEGF-A secreted by lung cancer cells in the TCM. As shown in Figure 4E–4H, the treatment with miR-140-3p and miR-140-5p, respectively, did not significantly change the secretion of VEGF-A in the TCM from the two cell lines (A549 and SK-MES-1) and at two time pointes (24 and 48 h).

### MiR-140-3p targets ITGB3

By combining the data from the main bioinformatics algorithms including TargetScan and MiRWalk, we identified integrin-β3 (ITGB3) as a potential direct target of miR-140-3p. In order to verify this canonical target, the DNA fragment of the wild-type ITGB3 3’-UTR (wt) which contains the 6-mer complementary with miR-140-3p and the mutated fragment of the ITGB3 3’-UTR (mut) were assembled into the pmirGLO vector, respectively.

**Figure 5:**
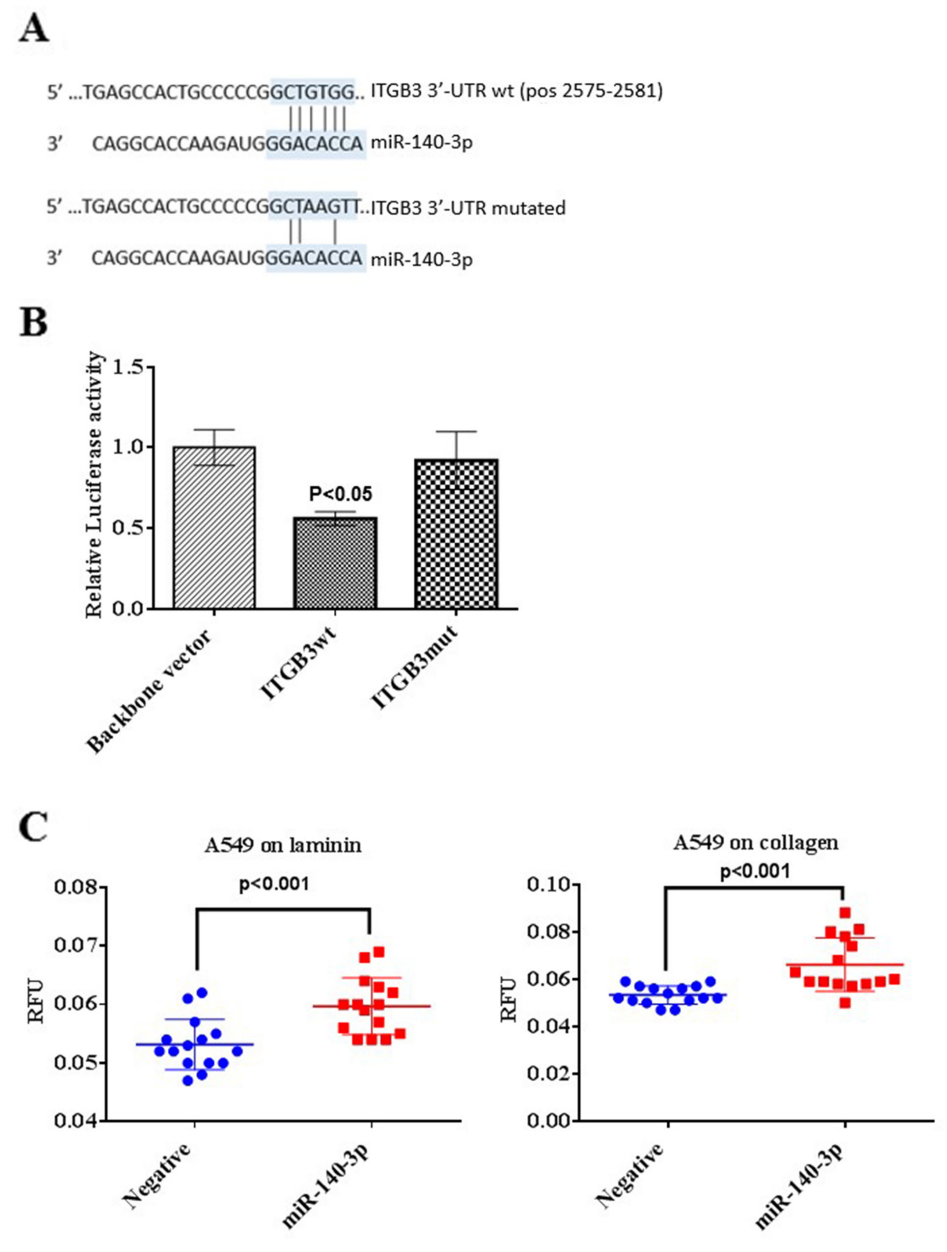
Validation of ITGB3 as a direct target of miR-140-3p **(A)** Predicted binding sites of ITGB3 3’-UTR and miR-140-3p using an online tool TargetScan. The base pairing between the ITGB3 3’UTR binding site and the seed region of miR-140-3p is showed by vertical lines (top). The binding site of miR-140-3p and the mutated form of ITGB3 3’-UTR includes 3 nucleotides (bottom). **(B)** Relative luciferase activity levels following 24 hours transfection of A549 cells with the Luciferase reporter vector containing the backbone vector only, ITGB3 3’-UTR (ITGB3wt) and the mutated form of the 3’-UTR (ITGB3mut), respectively. Data are presented as fold change of the mean + SEM, three individual experiments were undertaken in triplicate, ANOVA-test was used to assess significance with ^*^p<0.05. **(C)** Effect of miR-140-3p on adhesion ability of A549 cells on laminin and collagen type IV following transfection for 24 hours.

A549 cells containing the ITGB3wt resulted in a significant decrease of the luciferase activity when miR-140-3p were co-transfected compared to the vector control (p<0.05, Figure 5B). As a contrary, cells containing the ITGB3mut did not repress the activity of the luciferase, but, as expected, produced a signal similar to the negative control containing the vector only.

It is known that integrins participate in cell adhesion through its domain to bind ECM proteins, to indirectly confirm whether miR-140-3p may has an effect on cell adhesion through targeting ITGB3, adhesion ability of A549 cells was assessed after miR-140-3p treatment for 24 h. As shown in Figure 5C and 5D, miR-140-3p enhanced the adhesion property of A549 cells on laminin (p<0.001) and collagen (p<0.001), respectively compared to the control with the negative miRNA mimic.

### Two miRNA-140 strands play different roles in gene targeting and cell signalling pathways

**Figure 6:**
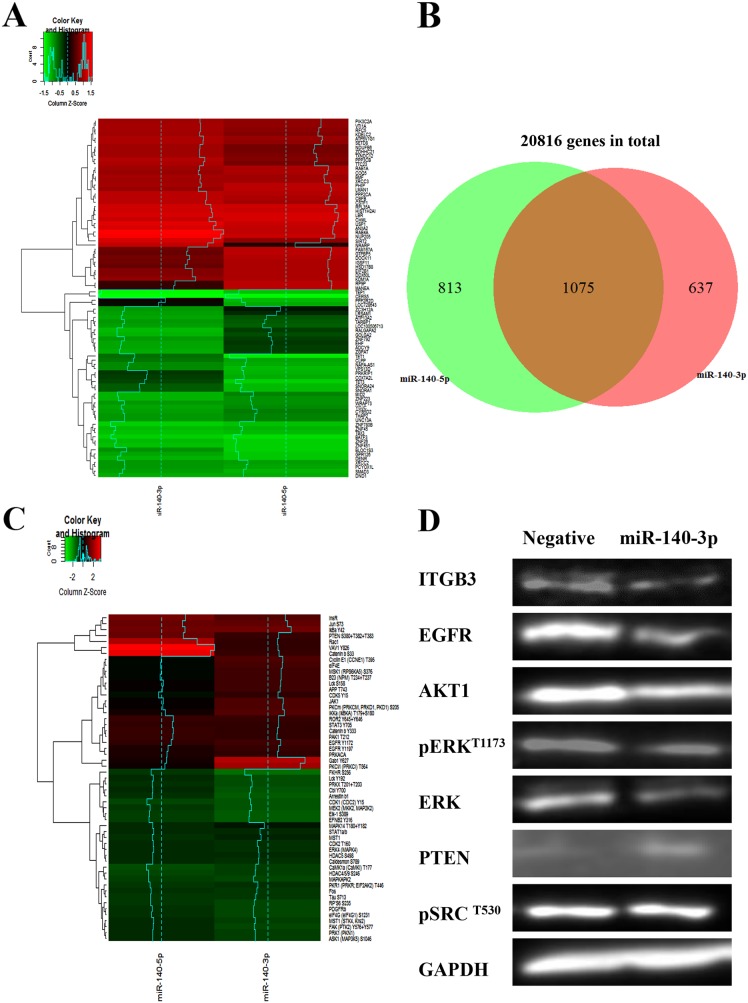
Distinctive gene regulation profiles and cell signalling pathways induced by two strands of miR-140 **(A)** Heatmap of the RNA-seq data showing the altered upregulated (red) and downregulated (green) genes in A549 treated with miR-140-3p and miR-140-5p, respectively. Global transcriptome analysis of 20816 gene targets was performed using the Ion AmpliSeq™ human gene expression panel. **(B)** The Venn diagram showing the differential gene expression induced by miR-140-3p and miR-140-5p, respectively, based on the RNA-Seq data. **(C)** Heatmap of the Kinex antibody array data showing the distinctive cell signalling pathways altered by the two strands of miR-140. The values of Z-score reflected positive or negative shifts in differential protein expression fold-changes after normalization by protein concentration and negative miRNA mimics. **(D)** Evaluation the expression of some key downstream effectors of miR-140-3p in A549 cells after treatments for 48 hours by Western blotting.

The integrated approaches of biotin-miRNA pulldown and transcriptome profiling by RNA-seq were used to screen the mRNA targets of the two miR-140 strands in a systematic manner [9]. As shown in Figure 6A, based on the criteria we have used, the RNA-Seq data indicate that there were 637 unique miR-140-3p targets and 813 unique miR-140-5p targets, and both miR-140 strands shared 1075 targets. When we zoomed in to compare the first and last 25 targets of each miR-140 strands, the RNA-Seq heatmap showed the different patterns of their targets. We further investigate the signalling pathways regulated by the two miR-140 strands using the Kinex Antibody Array which features 880 antibodies. As shown in Figure 6C, the heatmap indicated that two miRNA-140 regulated distinctive signalling patterns although some proteins were co-regulated.

We then attempted to integrate the data from RNA-Seq and antibody array in order to identify more significant targets. As shown in Table 1, six target candidates of miR-140-3p were identified, which included HDAC4, EGFR, FOS, IGF1R, RAC1 and CDK1. Within these targets CDK1 can be predicted by an *in silico* tool TarBase. Using the same criteria, we identified twelve miR-140-5p targets which included SMAD3, PTEN, MAPK12, PRKCE, IGF1R, INSR, FOX, IRS1, MAPK14 (p38α), JAK1, STAT3 and CAV1 (Table 2). Within this panel of targets, IGF1R was predicted by MIRTARBASE, while JAK1 and SMAD3 could be predicted by TARBASE. And IGF1R and FOS were targets of both miR-140-3p and miR-140-5p.

**Table 1:** Association of RNA-Seq data after biotinylated-miRNA pull down with Kinex™ Antibody Microarray data from cells treated with miR-140-3p

RNA-seq		Kinex™ Antibody Microarray		*In silico* prediction
Gene target	Log_2_(Fold change)	Protein phospho site	% (Change from control)	
HDAC4	-0.79	Pan-specific	-34.56	
EGFR	-0.67	Y1172	-55.69	
		Y1197	-52.00	
FOS	-0.44	T232	-33.27	
IGF1R	-0.40	Y1280	-33.81	
RAC1	0.44	S71	-38.49	
CDK1	0.51	Pan-specific	-37.13	TarBase

Data were normalized using cells treated with negative mimic control. Only the molecules which have a RNA-Seq value less than <-0.38 or over than 0.38, and an Antibody Microarray value <-30% or >30% were selected. The calculation of selection criteria is: log_2_(1.3) equivalent to 0.38. These miRNA target prediction/validation databases were searched: TarBase, miRTarBase, miRWalk, miRecords, miRTar, DIANA-microT, Microrna.org and Targetscan.

**Table 2:** Association of RNA-Seq data after biotinylated-miRNA pull down with Kinex™ Antibody Microarray data from cells treated with miR-140-5p

RNA-seq		Kinex™ Antibody Microarray		*In silico* prediction
Gene target	Log_2_(Fold change)	Protein phospho site	% (Change from control)	
SMAD3	-1.95	Pan-specific	-33.25	TARBASE
PTEN	-0.55	S380+T382+T383	-44.56	
MAPK12	-0.52	Pan-specific	-37.01	
PRKCE	-0.52	S729	-36.45	
IGF1R	-0.49	Y1280	-40.66	MIRTARBASE
		Y1165/Y1166	43.37	
INSR	-0.41	Y612	-47.59	
		Y1189/Y1190	-38.55	
		Y999	42.29	
FOS	-0.39	T232	-44.84	
		Pan-specific	53.27	
IRS1	0.40	S639	-32.35	
MAPK14	0.46	T180+Y182	-46.02	
JAK1	0.50	Y1034	-30.11	TARBASE
STAT3	0.56	Y705	-43.98	
CAV1	0.80	Pan-specific	-30.28	

Data were normalized using cells treated with negative mimic control. Only the molecules which have a RNA-Seq value less than <-0.38 or over than 0.38, and an Antibody Microarray value <-30% or >30% were selected. The calculation of selection criteria is: log2(1.3) equivalent to 0.38. These miRNA target prediction/validation databases were searched: TarBase, miRTarBase, miRWalk, miRecords, miRTar, DIANA-microT, Microrna.org and Targetscan.

### MiR-140-3p reduces the invasion properties of A549 cells and the angiogenic potential of endothelial cells through EGFR

There is evidence that ITGB3 may activate EGFR and p-Src in epithelial cells [10]. We further evaluated the protein levels of ITGB3 and EGFR signalling proteins in A549 cells. As shown in Figure 6D, Western blotting demonstrated the downregulation of ITGB3 after treatment with miR-140-3p in A549 cells. Furthermore, there was a decrease of EGFR protein level as well as its downstream checkpoint proteins AKT1 and ERK. However, there was a slight increase of PTEN, and the level of p-Src (Y530) remained unchanged.

## DISCUSSION

Previous clinical studies and our cohort screening data indicate that both miR-140-3p and miR-140-5p are downregulated in NSCLC and this is associated with the progression of the malignancy [1, 3, 4, 11]. Also, single-nucleotide polymorphism (SNP) or point mutation of the pre-miR-140 sequence may lead to the imbalanced formation of the two sister strands, for instance, an increase of miR-140-3p and decrease of miR-140-5p levels in the cleft palate syndrome, as the loss of targeting the Platelet-Derived Growth Factor-A (PDGFR-A) by miR-140-5p occurs [12]. Our previous study showed that treatment with miR-140-5p mimic can reduce the invasion properties of lung cancer cells by inhibiting the EGFR signalling pathway [1]. We herein further investigated the expression and gene targeting of miR-140-3p in lung cancer in comparison with its sister strand miR-140-5p in lung cancer.

Using the same lung cancer cohort, we observed that miR-140-3p is also downregulated in NSCLC like miR-140-5p. This observation is in line with the reports from other studies on miR-140-3p [13, 14]. Currently there is no report of the association of miR-130-3p with patient survival probably due to the lack of cohort with long follow up. However, lower has-miR-140-5p is linked with poorer overall survival of lung cancer patients (p<0.05) by an online tool for prognostic biomarker identification using the GEO and TCGA datasets (http://www.compbio.iupui.edu/progmir). Our data also indicate that the expression of these two strands of miR-140 in lung cancer cells is lower than that in normal lung epithelial cells BEAS-2B. In addition, in lung cancer cells, the expression of miR-140-3p is higher than miR-140-5p, suggesting that the mature strand formation of the pre-miR-140 in lung cancer cells is not even.

Using the miRNA mimics, we demonstrate that treatment with miR-140-3p suppresses the invasion of A549 and Sk-MES-1 lung cancer cells. This confirms the findings from other studies [4, 13]. However, our data indicate that miR-140-3p has no effect on migration, proliferation of lung cancer cells, which was unlike other studies [4, 13]. Also miR-140-3p does not induce any apoptosis and cell cycle arrest in lung cancer cells.

By metabolite analysis, we show that miR-140-3p and miR-140-5p demonstrate very little change in the intracellular metabolites within the cell lines, however a few metabolites levels were observed to have a small change in levels. In particular, glycine was shown to increase in the A549 cancer cell line following treatment with either mimic and miR-140-3p increases the level of one substance named as 18-bis(acetyloxy)Lanostane-7,11-dione, Interestingly, this latter metabolite substance has previously been reported to suppress tumor progression [15, 16]. Glycine has recently been reported as having a key role in cancer proliferation via cleavage of the amino acid by glycine decarboxylase and utilisation of the resulting one-carbon units to allow for increased mitochondrial activity [17, 18] and hence altered levels in cancer cells and an increase in A549 following treatment with miRNA mimics may reduce this metabolic pathway leading to elevations in uncatabolised glycine levels. Whilst other reports question the role of glycine decarboxylase in cancer proliferation, the same studies note that, in some tumours, glycine levels are depleted via the conversion of glycine to serine as an alternative pathway in which serine increases allow for proliferation [19]. The levels of malate appear to be elevated in the normal BEAS-2B cells compared to the cancer cell lines and this may represent the proposed role of increased cytosolic malate dehydrogenase in lung cancer patients [20] and the fact that treatment with either mimic does not cause an increase in malate levels compared to the normal lung cell lines, suggests that this metabolic contributor to cancer development is not affected by the miRNA mimics. Overall, however remarkably little change was seen at the metabolite level following the treatments utilised.

We also attempted to evaluate the ability of the endothelial cells HUVECs to form new vessels in response to the treatment of the cancer cells with miR-140-3p. The TCM from A549 cells treated with miR-140-3p for 24 hours reduced the tubule formation of the endothelial cells, respectively. The TCM medium from A549 cells treated with miR-140-5p for 48 hours also reduced the tubule formation of HUVEC cells, although it tended to be less potent than the miR-140-3p TCM groups. HUVEC cells also showed reduced levels of vessel-like mesh structure formation after treatment with the 24h-hour TCM of SK-MES-1 after treatment with miR-140-3p and miR-140-5p, respectively. It is known that quite often VEGF-A plays a crucial role in driving angiogenesis of endothelial cells particularly in pathological conditions. Our ELISA data however show that the VEGF-A secretion in TCM from both lung cancer cells was not altered after treatment with the two strands of miR-140 for 24 and 48 hours. Therefore, VEGF-A is not involved in the inhibitory effect of the TCM from lung cancer cells after treatment with the two strands of miR-140.

We then further investigated the potential targets of miR-140-3p in order to explain the functional alteration of lung cancer cells we have observed. By using a bioinformatics approach, we were able to identify ITGB3 as a direct target of miR-140-3p. It has been reported that integrins mediate the adhesion and migration properties of tumour cells by cooperating with specific growth factor receptors, such as EGFR [10, 21]. Also integrins are also expressed in tumour-associated cells, including endothelial cells [21]. There is evidence that integrins regulate various pathways depending on the stimuli in the tumour microenvironment [21]. In particular, ITGB3 is associated with the tumour progression [22]. For example, when ITGB3 is assembled with the α5 integrin subunit to form the α5β3 complex, it can regulate angiogenesis together with other growth factors receptors, such as the Fibroblast Growth Factor Receptor (FGFR) in a Src-independent manner [23]. After the validation of ITGB3 as a novel direct target of miR-140-3p, we further demonstrated that the transfection with miR-140-3p mimics enhanced the adhesion ability of A549 on laminin and collagen, two of the main components of the ECM. Also, the EMT process is halted by miR-140-3p mimics. During EMT, the tumour cells lose their epithelial phenotypes and acquire some mesenchymal-like properties that enable them to survive and form metastasis in distant sites [24]. We focused on some EMT markers specifically involved in lung cancer progression [25], and found that miR-140-3p restored the levels of E-cadherin while reduced the expression of N-cadherin, Vimentin, Slug and Snail. These findings could partly explain the alteration of migration and invasion of lung cancer cells with response to miR-140-3p.

It has been shown that miRNAs in the tumour microenvironment can be released by the cells encapsulated into either microvesicles or exosomes and internalised into the endothelial cells thus mediate angiogenesis [26–29]. We therefore speculate that miR-140-3p exerts an inhibitory effect on HUVECs by targeting ITGB3 for three main reasons. First of all, ITGB3 has been found up-regulated in both cancer and endothelial cells [21]. Secondly, the miRNAs in conditioned media from cancer cells after miR-140-3p mimic treatment may be incorporated in the endothelial cells. Finally, the inhibition of the tubule formation ability is VEGF-A independent and previous studies suggest the involvement of ITGB3 in angiogenesis [23].

To better understand the gene targeting and key signalling pathways mediated by the two strands of miR-140, we performed RNA-seq after biotin-miRNA pull down and Kinex Antibody Array. The RNA-seq data show that the gene targeting profiles of cells treated with the two forms of miR-140 are quite different although there is a certain level of overlapping target clusters. Likewise, the change of signalling pathways by two miR-140 isoforms is also distinguishable. By using a cut-off of 1.3-fold change, we were able to identify further targets of miR-140 isoforms after the integration of the two high throughput datasets of gene and signalling protein profiles. Our data show that the majority of these newly identified targets cannot be predicted by the conventional in-silico prediction tools. There are various potential reasons to explain this sort of as-expected mismatch [30, 31]. Firstly, the sequence complements of miRNA precursors are often not conserved thus beyond the capacity of our current computational prediction algorithms which highly relies on the conservation of sequence alignment. Secondly, non-canonical microRNA binding has been frequently observed, which also participates regulation of gene expression. In addition, integrated approaches of transcriptome and proteome data analysis present the advantage of identifying those more important or non-direct targets from complicated regulation and signalling networks. Despite the distinctive targeting or regulating profiles, the two strands of miR-140 share some targets such as IGF1R and FOS. Increased IGF-1R activity is implicated in tumorigenesis and cancer cell invasiveness and previous reports have shown that IGF1R is one of the targets of miR-140-5p. Our data show that miR-104-5p can also decrease gene expression and phospho-protein of IGF1R for the first time. The FOS gene has been found to overexpressed in several cancer types and considered as a proto-oncogene [32]. The FOS protein interacts with a panel of other proteins (c-Jun, junB, junD, FosB, Fra-1 and Fra-2) and forms the heterodimeric complex of Activator Protein-1 (AP-1) transcription factors which mediates multiple functions of cancer cells and is implicated as a driver of tumorigenesis [33].

**Figure 7:**
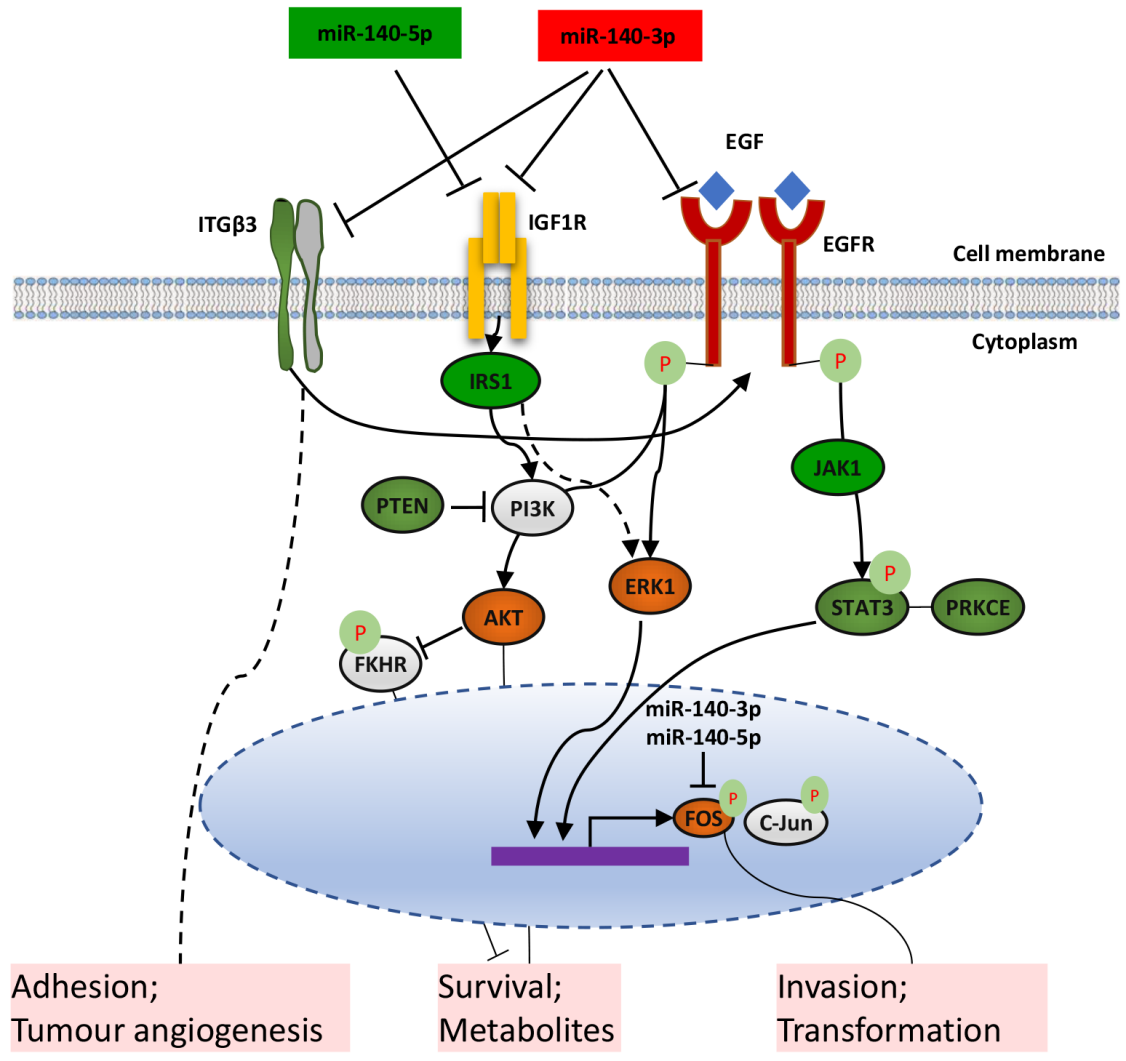
Schematic diagram of the proposed distinctive mechanisms that two forms of miR-140 suppress the metastatic properties of lung cancer cells

In combination with our data and literature, we speculate that the two strands of miR-140 show inhibitory effect on the behaviour of lung cancer cells in different mechanisms (Figure 7). miR-140-3p targets EGFR and IGF1R, which leads to the inactivation of the PI3K/AKT pathway which is involved in cancer cell survival and metabolism. Also, the ERK1 checkpoint in the downstream of EGFR and IGF1R is inactivated by miR-140-3p, leading to a reduction of active AP-1 proteins including FOS and c-JUN, thus modulate invasion and transformation of lung cancer cells. MiR-140-3p can also regulate STAT3 directly or indirectly through EGFR, which also mediates the expression of AP-1 transcription factors such as FOS and c-Jun. One of the other targets of miR-140-3p is ITGB3 which cooperates with EGFR as a complex and is also a key factor in tumour cell adhesion and tumour angiogenesis. In contrast, miR-140-5p can target IGF1R, leading to inactivation of the PI3K/AKT and an ERK1 axis through IRS1. miR-140-5p can also target PTEN to attenuate its suppression of the PI3K/AKT signalling, suggesting its multiple functions to regulate this pathway. Both strands of imR-140 can target FOS thus inhibit tumour cell invasion and modulate their transformation.

One of the limitations of this study is that the effect of ITGB3 downregulation by miR-140-3p needs to be further investigated in a tumor microenvironment containing other cell types such as stromal cells and fibroblasts. There is a report that ITGB3 expression is frequently induced by the transforming growth factor beta (TGFβ) secreted by the stromal cells, that acts as an inducer of the EMT [22, 34]. It will also be interesting to evaluate the therapeutic potential of the miR-140 strands in an *in vivo* model and by translational research in future.

In summary, we have demonstrated for the first time that miR-140-3p and miR-140-5p both suppress the malignant properties of lung cancer cells but through distinct and multiple mechanisms. This study provides novel insights into the roles of the two forms of pre-miR-140 products in lung cancer by taking advantage of the integrated approaches of RNA-seq after biotin-miRNA pull down and high-throughput antibody array.

## MATERIALS AND METHODS

### Patient tissue specimens

Fresh tissue samples from NSCLC patients were collected immediately after surgery and stored at -80°C until use by Capital Medical University Hospital, Beijing, China. The collection was approved from the Health Authority local research ethics committee. The recruited patients were informed and participated with a written consent. The cohort included 68 unpaired normal and tumour tissues with 19 paired normal and tumour lung tissues. All the specimens used in the current study were verified by a consultant pathologist. Tissues were divided in two categories, depending on the availability. Paired tissues refers to tissues from the same patients, in which the tumour part and the adjacent normal counterpart have been resected surgically, whereas the unpaired are tissues from different patients. The patient clinic-pathological information is described in Table 3.

**Table 3:** Clinicopathological characteristics of the non-small cell lung cancer (NSCLC) cohort

Characteristic		Number	Percentage	miR-140-3p p-value	miR-140-5p p-value
Gender	Female	28	41%	0.151	0.307
	Male	40	59%		
TNM	T1N1M0	1	1%	0.6992	0.810
	T1N2M0	2	3%		
	T1N2M1	10	15%		
	T2N2M0	11	16%		
	T2N3M0	1	1%		
	T3N0M0	1	1%		
	T3N2M0	3	4%		
	Unknown	39	57%		
Smoking history	Non-smokers	9	13%	0.110	0.851
	Smokers	34	50%		
	Unknown	25	37%		

It included 68 cases with 19 cases of paired tumour and adjacent-normal tissues. There were 68 NSCLC and 40 adjacent-normal tissues obtained from this cohort in total. The association of the expression of the two strands of miR-140 with the clinicopathological features was evaluated using Mann-Whitney *U* test (for Gender) or Kruskal-Wallis rank sum test (for TNM and Smoking history).

### Cell culture

A549 carcinoma and SK-MES-1 squamous carcinoma NSCLC cell lines were obtained from Lonza (Gloucestershire, UK) and maintained in Dulbecco's modified Eagle's medium (DMEM) with 10% foetal bovine serum (FBS) and antibiotics (100U/ml) (Gibco, Invitrogen, UK). The normal lung cell line BEAS-2B was maintained in Bronchial Epithelial Cell Growth Medium supplemented with BEGM™ Bullet Kit (Lonza, Slough, UK). The primers used for the evaluation of miRNA expression was listed in Table 4.

**Table 4:** Primers used for the evaluation of miRNA expression and miRNA target validation

Primer		Sequence
miR-specific RT		CAGGTCCAGTTTTTTTTTTTTTTTVN
RNU6B	Forward	GTCGTGAAGCGTTCCA
	Reverse	CAGGTCCAGTTTTTTTTTTTTTTTAAA
RNU48	Forward	ACCGCAGCGCTCT
	Reverse	TCCAGTTTTTTTTTTTTTTTGGTCA
miR140-5p	Forward	CAGCAGTGGTTTTACCCTATG
	Reverse	GGTCCAGTTTTTTTTTTTTTTTCTAC
miR140-3p	Forward	GTACCACAGGGTAGAACCA
	Reverse	GTACCACAGGGTAGAACCA
ITGB3-miR-140-3P	Forward	AAACTAGCGGCCGCTGAGCCACTGCCCCCGGCTGTGGTTGT
	Reverse	CTAGACAACCACAGCCGGGGGCAGTGGCTCAGCGGCCGCTAGTTT
ITGB3 mutated-miR-140-3P	Forward	AAACTAGCGGCCGCTGAGCCACTGCCCCCGGCTAAGTTGT
	Reverse	CTAGACAACTTAGCCGGGGGCAGTGGCTCAGCGGCCGCTAGTTT

In the sequences of the miR-specific RT primer, V = A or G or C, N = A or G or C or T. RNU6B and RNU48 were used for the normalization of the miRNA expression using a Sybr Green qRT-PCR miRNA assay. The predicted ITGB3 3’UTR target regions complementary to miR-140-3p was designed based on the prediction by the online tool TargetScan Human 7.0 (http://www.targetscan.org). The overhangs created by oligonucleotide annealing were designed to be complementary to those sites recognized by the restriction enzymes PmeI (GTTT^AAAC) and XbaI (T^CTAGA). NotI internal site (GC^GGCCGC) was added for cloning digestion.

### Transient miRNA transfection

Mimics of hsa-miR-140-3p, hsa-miR-140-5p and a scramble miR control were purchased from Sigma-Aldrich (Dorset, UK). A549 and SK-MES-1 cell lines were transiently transfected with these miRNA mimics using 2 μl/ml of DharmaFECT1 transfection reagent (GE Healthcare Dharmacon, Colorado, USA) in antibiotic-free medium.

### Quantitative real-time polymerase chain reaction (qRT-PCR)

Total RNA was extracted from all the cell lines and tissues using TRI reagent (Sigma-Aldrich, Dorset, UK) according to the manufacturer's instructions. The total RNA samples (500 ng) was converted into cDNA using a miRNA-compatible reverse transcription primer and the reverse transcription kit (New England BioLabs^®^, UK). The conditions used for reverse transcription were 42°C for 60 minutes and 95°C for 5 minutes using Applied Biosystems 2720 Thermal Cycler (Life Technologies, Paisley, UK). Following reverse transcription, cDNA was diluted 1:24 using DEPC water. PCR amplification was performed using the SYBR Green JumpStart Taq ReadyMix (Sigma-Aldrich, Dorset, UK) and specific reverse and forward primers for each miRNA (Sigma-Aldrich, Dorset) in a IQ5 Bio-Rad qPCR thermocycler. Reaction used the following parameters: pre-denaturation for 10 minutes at 94°C; followed by 40 cycles of denaturation at 94°C for 15 seconds, annealing and elongation at 58°C for 1 minute. Results were analysed using 2-(ΔΔCT) method of normalisation to housekeeping gene U6 or GAPDH in cell lines and U48 in tissues. A melting curve analysis was also included.

### Transwell invasion assays

Cells were detached using HYQTase Cell Detachment Solution (Hyclone, Logan, UT, USA) 24 hours after being transfected and resuspended in serum-free media, at a concentration of 1×10^5^ cells/ml. On the bottom of each well, 650 μl of medium containing 10% FCS (chemoattractant) or serum-free media (no chemoattractant) were added. Policarbonate 8μm pore sized ThinCert™ 24-well plate inserts (Grenier, Bio-One GmbH, Austria) were placed in each well and filled with 500 μl of cell suspension. Chambers were coated with serum-free medium containing Matrigel (BD Biosciences, NJ USA) to gain a concentration of 500μg/ml. After 24 hour incubation at 37°C, the inserts were washed with PBS and then incubated 1 hour at 37°C in 350μl Cell Dissociation Solution (CDS; Sigma Aldrich, Dorset, UK)/Calcein AM (eBioscience, Hatfield, UK) at a ratio of 1.2 μl Calcein AM in 1 ml CDS. The cells suspension was then transferred into a black 96-well plate and fluorescence was measured (excitation 485nm/emission 520nm) using the Glomax Multi Detection System (Promega, Wisconsin, USA). To analyse the total directed cell movement, the wells containing no chemoattractant was subtracted from the test wells.

### Adhesion assay

A total of 1×10^4^ transfected cells/well were added into a 96 well plate coated with 100 μg/well of either laminin or collagen. A549 and SK-MES1 were allowed to attach at 37°C for 40 and 60 minutes, respectively, then washed with PBS, fixed using 200 μl of 4% Formalin and incubated at room temperature for 15 mins. Crystal Violet 0.5% was then added into each well, the plate incubated again at room temperature for 10 minutes, washed twice with distilled water and finally left overnight at 37°C to dry. The following day the absorbance was measured using the Glomax Multi Detection System (Promega, Wisconsin, USA).

### Tubule formation assay

A total of 1.4×10^5^ HUVEC cells/ml in complete medium and 100 μl of either A549 or SK-MES1 medium were added to each well of a 96-well plate coated with serum-free medium containing 50% Matrigel (BD Biosciences, NJ USA). 100 μl of 60% diluted tumour condition medium from cells treated with miRNA mimics was added to each well and the tubule formation was monitored for 6 hours in time lapse using the EVOS System (Thermo Fisher Scientific, Waltham, MA USA).

### Enzyme linked immunosorbent assay (ELISA)

The human vascular endothelial growth factor A (VEGF-A) levels in the TCM of A549 cells transfected with negative and miR-140-3p mimics were quantified by using 96-well plates pre-coated with VEGF-A primary antibody by using a specific kit purchased from Aviva System Biology, San Diego, USA. Following 24 hours of negative and miR-140-3p treatment, the media was transferred in a new tube and centrifuged at 1000 xg for 15 minutes to remove any cellular debris. The samples were then processed following the manufacturer's instructions. The absorbance was measured using Glomax Multi Detection System (Promega, Wisconsin, USA).

### *In silico* analysis of miR-140-3p and its targets

To evaluate the putative mRNA targets of miR-140-3p and miR-140-5p, computational methods (Diana mT, miRanda, miRWalk, TargetScan) were used.

### MiRNA target validation by using *in silico* tools

The pmirGLO Dual-Luciferase miRNA Target Expression Vector (Promega, Wisconsin, USA) was used to evaluate miRNA activity by inserting the putative miRNA target sites 3′ of the firefly luciferase gene (luc2). The target sites were cloned into the pmiRGLO Vector, propagated in the One Shot^®^ TOP10 Chemically Competent E. coli cells (Invitrogen, Carlsbad, CA) and purified using GenElute™ Plasmid Miniprep Kit (Sigma-Aldrich, Dorset, UK). Plasmids were partially sequenced to verify the integrity of the cloned inserts. Cells were transfected with plasmids containing the pmiRGlo vector and wild-type and mutated target sequences of miR-140-3p. After 24 hours cells were analysed for luciferase activity using the Dual-Glo^®^ Luciferase Assay System (Promega, Wisconsin, USA) and the Glomax Multi Detection System (Promega, Wisconsin, USA. Normalised firefly luciferase activity (Firefly/Renilla) for each construct was compared to that of the pmiRGLO vector no-insert control. The primers used for the miRNA target validation was listed in Table 4 as well.

### Protein microarray (Kinexus)

Following 48 hours of treatment of A549 with negative, miR-140-3p and miR-140-5p miRNA mimics, cells were lysed as indicated in Section 2.14 and sent to Kinexus Bioinformatic Corporation for the analysis. Results were sorted in Excel by the lowest % CFC (percentage change from control) in response to the miRNA treatment.

### Western blotting

Following 48 hours of treatment with miR-140-5p mimics, cells were lysed using RIPA buffer. The protein concentration was determined, an equal volume of 2x Llamelli buffer (Sigma Aldrich, Dorset, UK) was added and the mixture was heated to 95°C for 5 minutes, then loaded on to a gel. Gel was run for 2.5 hours at 100V. Gel was transferred onto PVDF membrane using wet transfer for 50 minutes, at 500mA. The membrane was blocked in 5% milk for 1 hour, and probed overnight with the primary antibodies. All the primary antibodies used in this study were purchased from Santa Cruz Biotechnology (Santa Cruz, USA). All the antibodies were diluted 1:200, except GAPDH that was diluted 1:1000 Membranes were washed three times in TBS-Tween (0.01%) and then probed with the appropriate secondary antibody purchased from Sigma-Aldrich (Dorset, UK) with a dilution of 1:1000. Protein bands were detected using EZ-ECL (Biological Industries, Staffordshire, UK) and visualised using a G:BOX (Syngene, Cambridge, UK).

### Gas chromatography–mass spectrometry (GC-MS)

Cells were treated with the microRNA for 24 hours and then cells pelleted and stored at -80˚C prior to analysis. For metabolite extraction, the cell pellets were sonicated for 15 minutes after the addition of 500μL of ice cold methanol. The supernatant was taken and dried to dryness in a vacuum centrifuge. All dried samples were then derivatized by addition of 30 μL methoxylamine hydrochloride (15 mg/mL in pyridine), followed by incubation at 70°C for 60 min. 50 μL of MSTFA (Thermo scientific) was then added. The samples were then heated at 40°C for a further 90 min. Finally, 10 μL of internal standard tetracosane (2 mg/1 mL in heptane) was added to each vial prior to GC–MS analysis. The samples were analysed by injection of 1μl of sample onto an Agilent HP5975C GC-MS using a Durabond DB-5 column (Length- 30m, diameter 0.250mm with a 0.25um film) and a helium carrier gas. A temperature gradient was applied holding at 50°C for 2 minutes followed by an increase to 300°C at a rate of 10°C per minute. The GCMS utilised an electron impact ion source with an emission voltage of 70eV, single quadrupole ion separation and detection between 50-600amu. The chromatograms generated by the GC-MS were then statistically compared and the significant compounds identified. All peak areas were manually aligned before normalisation to the internal standard and metabolites were identified by comparison to the NIST library database.

### Statistical analysis

D’Agostino-Pearson test was used to verify if the data were normally distributed. Unpaired or paired *t*-test was used for data with normal distributions, whereas for non-normal distributions, the Mann-Whitney Rank Test was applied. When more than two sets of data were compared, either One-Way ANOVA or the non-parametric Kruskal-Wallis test was used. Graphs and the statistical analysis were performed using GraphPad Prism 6.04 software (GraphPad Software, San Diego, CA, USA). Statistical significance was indicated with the following nomenclature: ^*^p<0.05, ^**^p<0.01, ^***^p<0.001.

## SUPPLEMENTARY MATERIALS FIGURES AND TABLES



## References

[1] Flamini V, Jiang WG, Cui Y (2017). Therapeutic Role of MiR-140-5p for the Treatment of Non-small Cell Lung Cancer. Anticancer Res.

[2] Schoen C, Aschrafi A, Thonissen M, Poelmans G, Von den Hoff JW, Carels CE (2017). MicroRNAs in Palatogenesis and Cleft Palate. Front Physiol.

[3] Yuan Y, Shen Y, Xue L, Fan H (2013). miR-140 suppresses tumor growth and metastasis of non-small cell lung cancer by targeting insulin-like growth factor 1 receptor. PLoS One.

[4] Dong W, Yao C, Teng X, Chai J, Yang X, Li B (2016). MiR-140-3p suppressed cell growth and invasion by downregulating the expression of ATP8A1 in non-small cell lung cancer. Tumour Biol.

[5] Tilli TM, Bellahcène A, Castronovo V, Gimba ER (2014). Changes in the transcriptional profile in response to overexpression of the osteopontin-c splice isoform in ovarian (OvCar-3) and prostate (PC-3) cancer cell lines. BMC Cancer.

[6] Li Q, Yao Y, Eades G, Liu Z, Zhang Y, Zhou Q (2014). Downregulation of miR-140 promotes cancer stem cell formation in basal-like early stage breast cancer. Oncogene.

[7] Ferlay J, Soerjomataram I, Dikshit R, Eser S, Mathers C, Rebelo M, Parkin DM, Forman D, Bray F (2015). Cancer incidence and mortality worldwide: sources, methods and major patterns in GLOBOCAN 2012. Int J Cancer.

[8] Lee MH, Lahusen T, Wang RH, Xiao C, Xu X, Hwang YS, He WW, Shi Y, Deng CX (2012). Yin Yang 1 positively regulates BRCA1 and inhibits mammary cancer formation. Oncogene.

[9] Thomson DW, Bracken CP, Goodall GJ (2011). Experimental strategies for microRNA target identification. Nucleic Acids Res.

[10] Bill HM, Knudsen B, Moores SL, Muthuswamy SK, Rao VR, Brugge JS, Miranti CK (2004). Epidermal growth factor receptor-dependent regulation of integrin-mediated signaling and cell cycle entry in epithelial cells. Mol Cell Biol.

[11] Li W, He F (2014). Monocyte to macrophage differentiation-associated (MMD) targeted by miR-140-5p regulates tumor growth in non-small cell lung cancer. Biochem Biophys Res Commun.

[12] Li L, Meng T, Jia Z, Zhu G, Shi B (2010). Single nucleotide polymorphism associated with nonsyndromic cleft palate influences the processing of miR-140. Am J Med Genet A.

[13] Kong XM, Zhang GH, Huo YK, Zhao XH, Cao DW, Guo SF, Li AM, Zhang XR (2015). MicroRNA-140-3p inhibits proliferation, migration and invasion of lung cancer cells by targeting ATP6AP2. Int J Clin Exp Pathol.

[14] Tan X, Qin W, Zhang L, Hang J, Li B, Zhang C, Wan J, Zhou F, Shao K, Sun Y, Wu J, Zhang X, Qiu B (2011). A 5-microRNA signature for lung squamous cell carcinoma diagnosis and hsa-miR-31 for prognosis. Clin Cancer Res.

[15] Arata S, Watanabe J, Maeda M, Yamamoto M, Matsuhashi H, Mochizuki M, Kagami N, Honda K, Inagaki M (2016). Continuous intake of the Chaga mushroom (Inonotus obliquus) aqueous extract suppresses cancer progression and maintains body temperature in mice. Heliyon.

[16] Nakata T, Yamada T, Taji S, Ohishi H, Wada S, Tokuda H, Sakuma K, Tanaka R (2007). Structure determination of inonotsuoxides A and B and in vivo anti-tumor promoting activity of inotodiol from the sclerotia of Inonotus obliquus. Bioorg Med Chem.

[17] Chen G, Lucas S, Wang J (2014). Glycine decarboxylase is a target for transcriptional repressor Snail. Cancer Metab.

[18] Jain M, Nilsson R, Sharma S, Madhusudhan N, Kitami T, Souza AL, Kafri R, Kirschner MW, Clish CB, Mootha VK (2012). Metabolite profiling identifies a key role for glycine in rapid cancer cell proliferation. Science.

[19] Labuschagne CF, van den Broek NJ, Mackay GM, Vousden KH, Maddocks OD (2014). Serine, but not glycine, supports one-carbon metabolism and proliferation of cancer cells. Cell Reports.

[20] Zhang B, Tornmalm J, Widengren J, Vakifahmetoglu-Norberg H, Norberg E (2017). Characterization of the Role of the Malate Dehydrogenases to Lung Tumor Cell Survival. J Cancer.

[21] Desgrosellier JS, Cheresh DA (2010). Integrins in cancer: biological implications and therapeutic opportunities. Nat Rev Cancer.

[22] Hong SK, Park JR, Kwon OS, Kim KT, Bae GY, Cha HJ (2016). Induction of integrin β3 by sustained ERK activity promotes the invasiveness of TGFβ-induced mesenchymal tumor cells. Cancer Lett.

[23] Hood JD, Frausto R, Kiosses WB, Schwartz MA, Cheresh DA (2003). Differential alphav integrin-mediated Ras-ERK signaling during two pathways of angiogenesis. J Cell Biol.

[24] Zeisberg M, Neilson EG (2009). Biomarkers for epithelial-mesenchymal transitions. J Clin Invest.

[25] Heerboth S, Housman G, Leary M, Longacre M, Byler S, Lapinska K, Willbanks A, Sarkar S (2015). EMT and tumor metastasis. Clin Transl Med.

[26] Arroyo JD, Chevillet JR, Kroh EM, Ruf IK, Pritchard CC, Gibson DF, Mitchell PS, Bennett CF, Pogosova-Agadjanyan EL, Stirewalt DL, Tait JF, Tewari M (2011). Argonaute2 complexes carry a population of circulating microRNAs independent of vesicles in human plasma. Proc Natl Acad Sci U S A.

[27] Zhou W, Fong MY, Min Y, Somlo G, Liu L, Palomares MR, Yu Y, Chow A, O’Connor ST, Chin AR, Yen Y, Wang Y, Marcusson EG (2014). Cancer-secreted miR-105 destroys vascular endothelial barriers to promote metastasis. Cancer Cell.

[28] Turchinovich A, Cho WC (2014). The origin, function and diagnostic potential of extracellular microRNA in human body fluids. Front Genet.

[29] Turchinovich A, Samatov TR, Tonevitsky AG, Burwinkel B (2013). Circulating miRNAs: cell-cell communication function?. Front Genet.

[30] Afonso-Grunz F, Müller S (2015). Principles of miRNA-mRNA interactions: beyond sequence complementarity. Cell Mol Life Sci.

[31] Oulas A, Karathanasis N, Louloupi A, Pavlopoulos GA, Poirazi P, Kalantidis K, Iliopoulos I (2015). Prediction of miRNA targets. Methods Mol Biol.

[32] Schönthal A, Herrlich P, Rahmsdorf HJ, Ponta H (1988). Requirement for fos gene expression in the transcriptional activation of collagenase by other oncogenes and phorbol esters. Cell.

[33] Eferl R, Wagner EF (2003). AP-1: a double-edged sword in tumorigenesis. Nat Rev Cancer.

[34] Ischenko I, Liu J, Petrenko O, Hayman MJ (2014). Transforming growth factor-beta signaling network regulates plasticity and lineage commitment of lung cancer cells. Cell Death Differ.

