# Supplementary material for: Distinct mechanisms by which two forms of miR-140 suppress the malignant properties of lung cancer cells
Source: Oncotarget. 2018 Nov 23;9(92):36474–91. doi: 10.18632/oncotarget.26356 (PMC6284864; doi:10.18632/oncotarget.26356)
Supplement: Supplementary file 1 [file oncotarget-09-36474-s001.pdf]

Distinct mechanisms by which two forms of miR-140 suppress the malignant properties of lung cancer cells

SUPPLEMENTARY MATERIALS

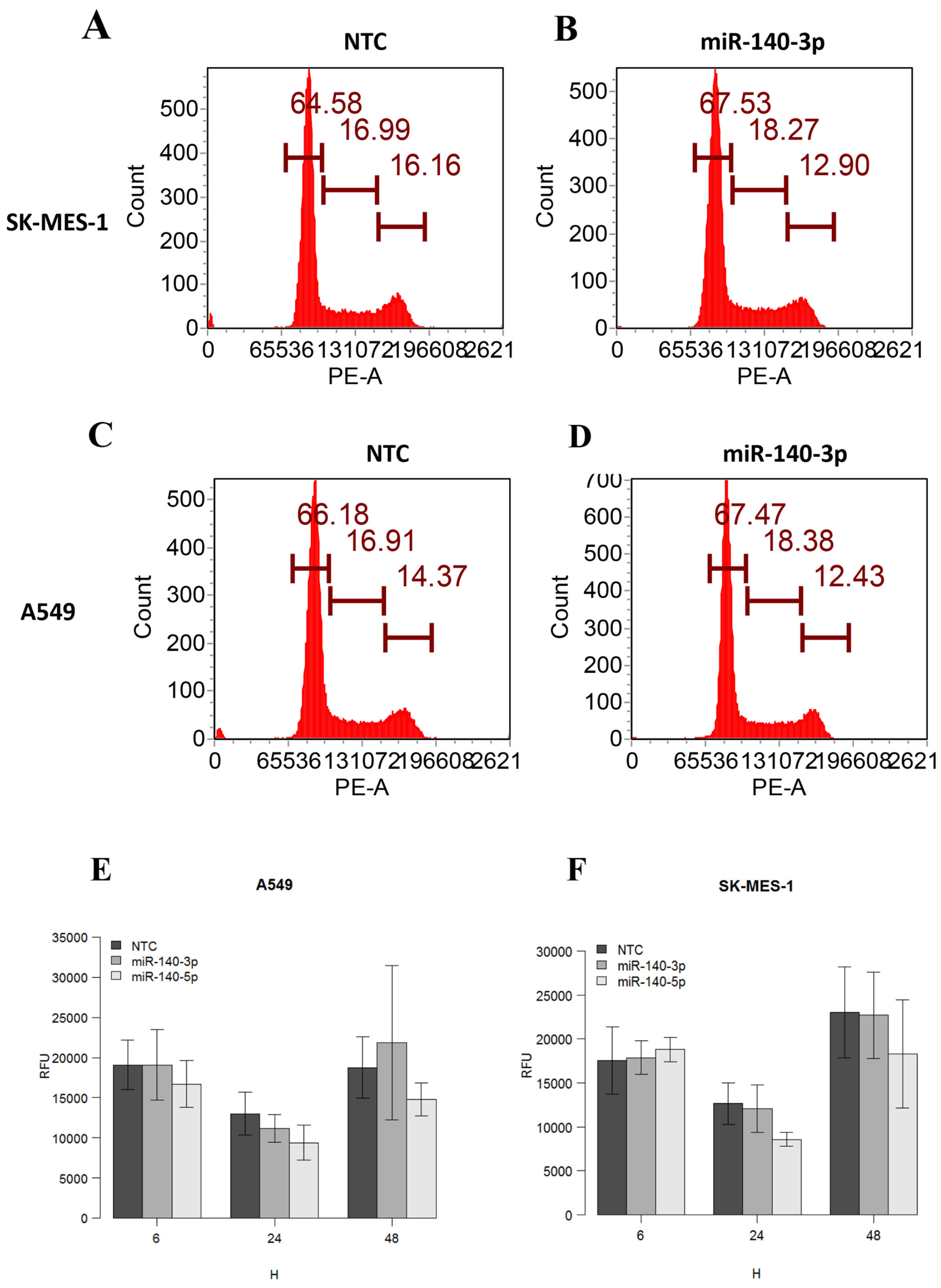

Supplementary Figure 1: Evaluation of cell cycle and proliferation of lung cancer cells treated with miR-140 mimics. **(A-D)** Cell cycle analysis after treatments for 48 hours. **(E-F)** Relative fluorescence unit (RFU) from Alamar Blue assay indicating the proliferation levels of cells.

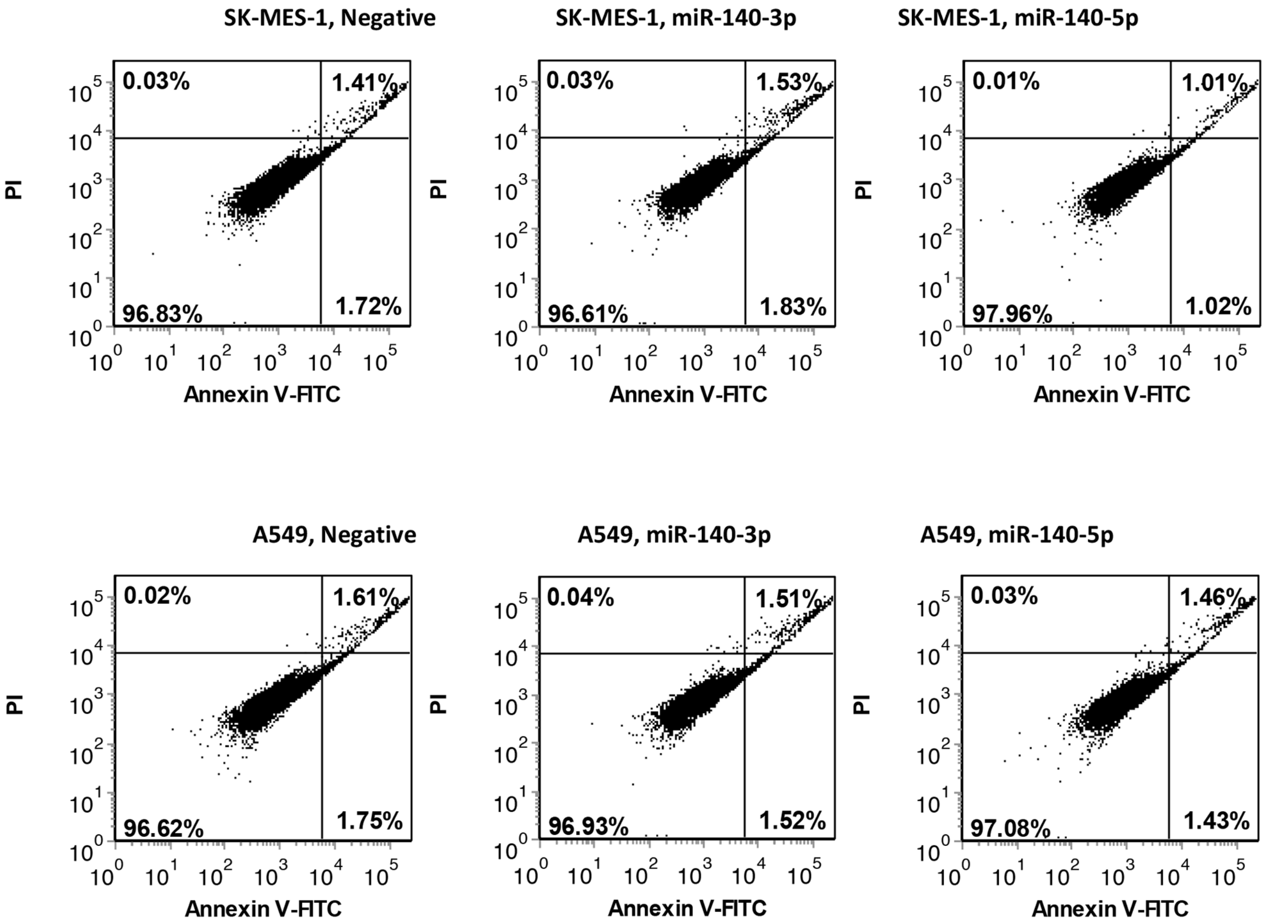

Supplementary Figure 2: Apoptosis analysis of lung cancer cells treated with miR-140 mimics.

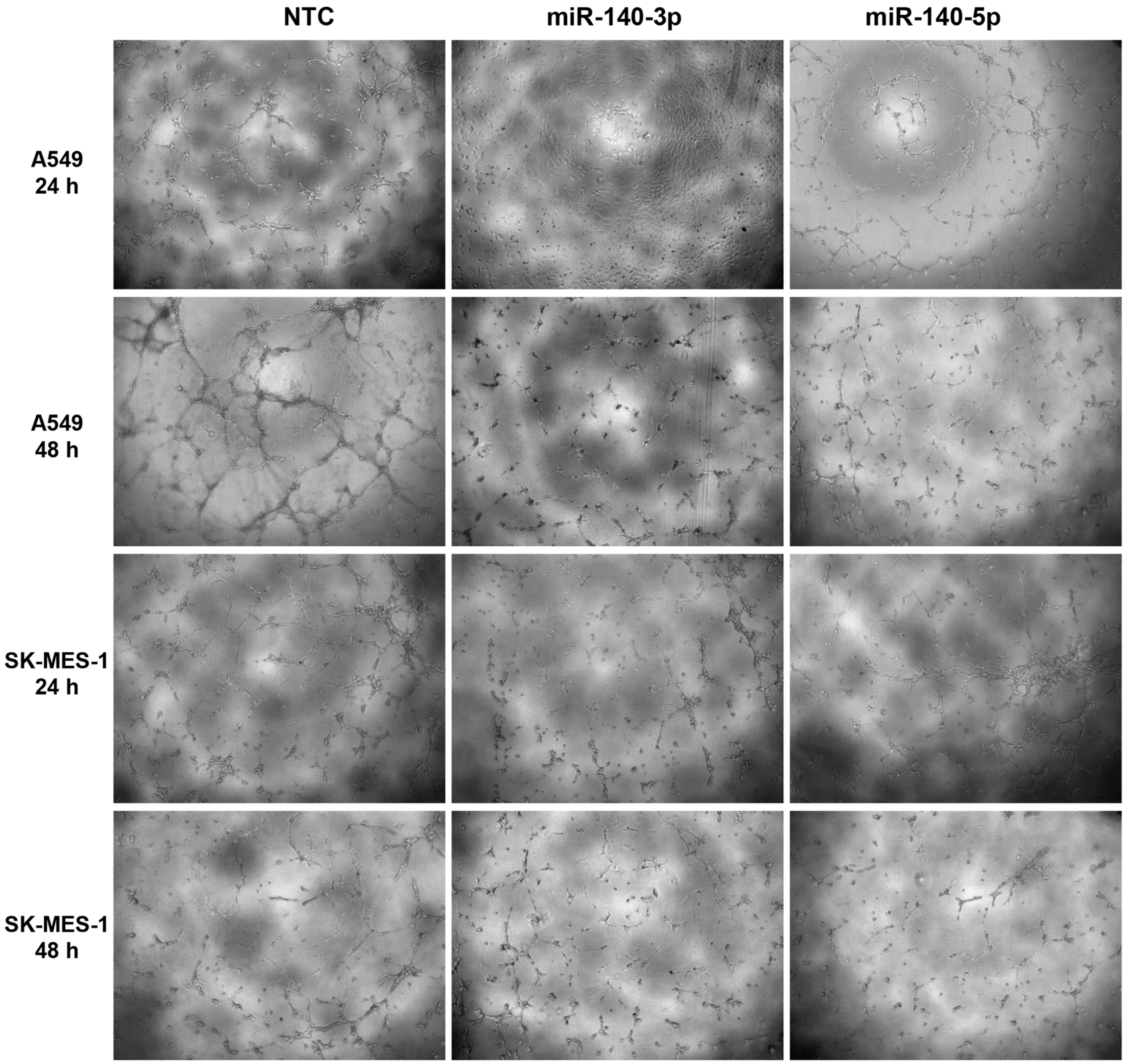

Supplementary Figure 3: Representative images of the tubule formation of the HUVECs co-cultured with TCM of lung cancer cells treated with miR-140-3p and 140-5p mimics.
